# Serious complications and risk of re-operation after Dupuytren’s disease surgery: a population-based cohort study of 121,488 patients in England

**DOI:** 10.1038/s41598-020-73595-y

**Published:** 2020-10-05

**Authors:** Osaid Alser, Richard S. Craig, Jennifer C. E. Lane, Albert Prats-Uribe, Danielle E. Robinson, Jonathan L. Rees, Daniel Prieto-Alhambra, Dominic Furniss

**Affiliations:** 1 Oxford NIHR Musculoskeletal Biomedical Research Unit, Nuffield Department of Orthopaedics, Rheumatology and Musculoskeletal Sciences, Nuffield Orthopaedic Centre, Botnar Research Centre, University of Oxford, Windmill Road, Oxford, OX3 7LD UK; 2 Centre for Statistics in Medicine, NDORMS, University of Oxford, Windmill Road, Oxford, OX3 7LD UK; 3 Department of Plastic and Reconstructive Surgery, Nuffield Orthopaedic Centre, Windmill Road, Oxford, OX3 7HE UK

**Keywords:** Musculoskeletal system, Musculoskeletal abnormalities, Epidemiology

## Abstract

Dupuytren’s disease (DD) is a common fibro-proliferative disorder of the palm. We estimated the risk of serious local and systemic complications and re-operation after DD surgery. We queried England’s Hospital Episode Statistics database and included all adult DD patients who were surgically treated. A longitudinal cohort study and self-controlled case series were conducted. Between 1 April 2007 and 31 March 2017, 121,488 adults underwent 158,119 operations for DD. The cumulative incidence of 90-day serious local complications was low at 1.2% (95% CI 1.1–1.2). However, the amputation rate for re-operation by limited fasciectomy following dermofasciectomy was 8%. 90-day systemic complications were also uncommon at 0.78% (95% CI 0.74–0.83), however operations routinely performed under general or regional anaesthesia carried an increased risk of serious systemic complications such as myocardial infarction. Re-operation was lower than previous reports (33.7% for percutaneous needle fasciotomy, 19.5% for limited fasciectomy, and 18.2% for dermofasciectomy). Overall, DD surgery performed in England was safe; however, re-operation by after dermofasciectomy carries a high risk of amputation. Furthermore, whilst serious systemic complications were unusual, the data suggest that high-risk patients should undergo treatment under local anaesthesia. These data will inform better shared decision-making regarding this common condition.

## Introduction

Dupuytren’s disease (DD) is a very common fibro-proliferative condition that results in thickening of the palmar fascia and flexion deformity of fingers^1^. In the UK, the estimated prevalence of DD varies from 0.2 to 30% depending on age, with wide geographical variation^2^. DD usually affects a more elderly population and it is more commonly seen in males than females^3^. DD causes disability, loss of hand function, and adversely affects quality of life^4^.

Although new non-surgical treatment modalities have been introduced in the last few years, the mainstay of treatment is still surgery^5^. The most commonly described surgical options for treatment of DD are percutaneous needle fasciotomy (PNF) and open surgical procedures such as limited fasciectomy (LF) and dermofasciectomy (DF).

As with any surgical procedure, DD surgery carries potential risks of local and systemic complications, in addition to the risk of recurrence requiring re-operation following primary surgery. Very few large-scale studies have evaluated the risk of postoperative complications or re-operations. For instance, in a survey-based chart review study from 12 European counties, Bainbridge et al.^6^ reported an overall postoperative complication rate of 23% in 3357 patients treated surgically for DD. In a series of 253 DD patients treated with LF, Bulstrode et al.^7^ found a complication rate of 18%, of which three patients suffered a systemic complication, namely urinary retention, myocardial infarction, and congestive heart failure. To our knowledge, no other published studies have examined the incidence of systemic complications following DD surgery.

There is a clear need for well-powered multi-centre studies to investigate the risk of complications and re-operation following surgery for DD, in order to better inform patients and clinicians of the risks and benefits of surgery. This was highlighted by the recent Priority Setting Partnership between the British Society for Surgery of the Hand and the James Lind Alliance^8^, where patients, carers and clinicians prioritised the research question “In patients with Dupuytren's disease, what invasive techniques give the best results in terms of function, recurrence and cost?”.

The primary aim of this study was to estimate the cumulative incidence of serious local and serious systemic complications 30 and 90 days after DD surgery and to determine the relative risk of serious systemic complications in the postoperative period using a self-controlled case series design. The secondary aim was to evaluate the rate and determinants of re-operation following primary DD surgery.

## Methods

### Study design and setting

We conducted a longitudinal nationwide population-based cohort study and self-controlled case series using Hospital Episode Statistics (HES) for National Health Service (NHS) England linked with the Office for National Statistics (ONS) mortality data.

### Participants

Our study population consisted of any surgical procedure for Dupuytren's disease undertaken between 1 April 2007 and 31 March 2017 in a patient over 18 years of age. We extracted information from the Admitted Patient Care (APC) data in the HES dataset. Operations with missing side code and operations that had less than 90 days follow-up after surgery (total n = 7286; 4.6%) were excluded from the analysis of local complications. For systemic complications, only operations with less than 90 days follow-up after surgery (n = 4743; 3%) were excluded from the analysis. Operations with missing laterality code (n = 2577; 1.63%) were excluded from the analysis of re-operation rate.

### Exposure

The exposure was DD surgical procedures. These operations were grouped into percutaneous needle fasciotomy (PNF), limited fasciectomy (LF) and dermofasciectomy (DF). Surgeries were identified using a pre-specified list of the International Classification of Diseases, 10th revision (ICD-10) codes and the 4th edition of the Office of Population Census and Survey (OPCS-4) codes (Supplementary Table 1).

### Outcomes

The outcomes were any serious local or systemic adverse events, which were identified using ICD-10 and OPCS-4 codes (see Supplementary Table 2a,b). Serious local complications were defined as surgical site infection (SSI) requiring wound debridement, wound dehiscence requiring surgical closure, neurovascular and tendon injury requiring surgical repair, and finger amputation that occurred in the operated hand. SSI and wound dehiscence were also combined into one category “wound complications”. Serious systemic complications studied were stroke, lower respiratory tract infection (LRTI), acute myocardial infarction (AMI), pulmonary embolism (PE), urinary tract infection (UTI), acute kidney injury (AKI), and death.

Re-operation after DD surgery was a secondary outcome. Within OPCS, revision procedure codes are only available for revision LF and revision DF. Because finger specific codes are not available in OPCS, someone having a second operation on the same hand that was coded as a primary procedure in OPCS might be undergoing true primary surgery on a different finger, or true revision surgery following a primary procedure. This limitation of the OPCS coding system prevents analysis of the number of procedures per finger, and therefore we used the term 're-operation' to describe any further surgery on the same hand following primary surgery.

### Data source

We analysed a bespoke extract from prospectively collected APC data in the HES dataset. The HES APC data extract contained all incident and prevalent episodes of admitted care for all adult patients at all National Health Services (NHS) hospitals, including NHS treatment provided in the independent sector, in England with at least one DD treatment episode between 1st April 2007 and 31st March 2017. This dataset was linked with the ONS mortality data for the purpose of survival analysis in this study.

### Study size

The final HES APC extract included 158,119 procedures performed in 121,488 patients.

### Statistical methods

#### Cumulative incidence of postoperative complications

Cumulative (unadjusted) incidence was calculated, and 95% confidence intervals (CI) were estimated using the Rothman and Greenland method^9^, for serious local and systemic complications within 30 and 90 days after all DD surgery. We selected 30 and 90 days after surgery as recommended by the NHS Outcome Framework guideline^10^. Only complications severe enough to require re-admission to hospital were included, as these were coded in HES APC.

#### Self-controlled case series

**Figure 1:**
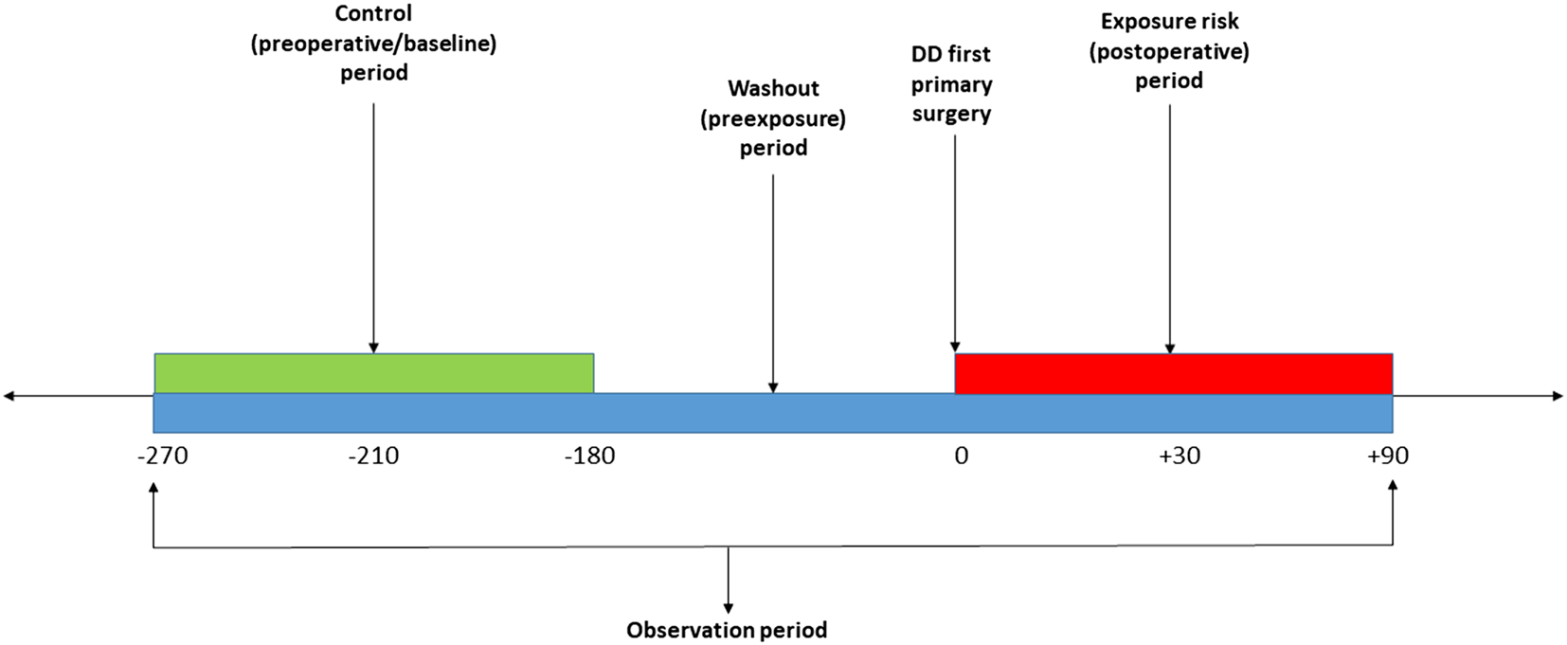
Schematic representation of the self-controlled Case Series (SCCS) model showing exposure risk period (red), control or baseline period (green), washout period and observation periods (dark blue).

We used a self-controlled case series (SCCS) study design (Fig. 1) to estimate the relative risk of serious systemic complications in the first 30 and 90 days following surgery. This design is a within-case comparison, requiring no separate comparator: each case acts as its own control to eliminate any time constant confounders^11,12^. We further hypothesised that procedures generally performed under general or regional anaesthesia (LF or DF) would increase the risk of serious systemic complications more than those routinely carried out under local anaesthesia (PNF). Our analysis was limited to the first primary DD surgery as studying beyond the first primary DD surgery could result in an overlap between an exposure period of one surgery and a baseline period of another surgery.

The baseline observation period was defined, for 90-day adverse events, as any time between 270 and 180 days before surgery (Fig. 1), and for 30-day events from 210 to 180 days before surgery**.** The rate of serious systemic events that occurred within 30 or 90 days after surgery, the exposure risk period, were then compared with their rate in each corresponding baseline risk period. A washout (pre-exposure) period including the 6 months before surgery was used to comply with one of the key assumptions of the SCCS, that the occurrence of an adverse event should not affect the probability of exposure^13^. In this case, the occurrence of any serious systemic adverse event would temporarily decrease the probability of having DD surgery.

Less than 1% of patients in our cohort died after surgery prior to experiencing any systemic adverse event, so we did not account for death in our analysis. As age is considered a time-varying confounder, it was adjusted for by including it in the SCCS model. Age was categorised into bands in order for the underlying Poisson model to accommodate rare events. Age categories for most complications were 40–59 years, 60–64, 65–69, 70–74, 75–79, 80–84, 85–89, 90–94 and 95 and above. For two complications, AKI and PE, different age categories were used due to a lower incidence of complications in younger and older patients, and hence a lack of model convergence.

Conditional Poisson regression offset by the natural logarithm of risk intervals was used to study the association between exposure to surgery and the occurrence of serious adverse events within 30 or 90 days after surgery. The incidence rate ratio (IRR) and 95% CI were calculated for each adverse event. Interactions with gender were examined by adding this variable to the SCCS model as a multiplicative interaction. The analysis was also repeated with the case cohorts stratified by type of surgery.

### Survival analysis

Kaplan Meier survival estimates were used to determine the cumulative probability of re-operation after primary surgery. The analysis was limited to the first re-operation following all primary DD surgery, and for each surgical subtype. Multivariable Cox proportional hazards regression analysis was used to estimate the hazard ratio (HR) and 95% confidence interval (CI) for each potential determinant of re-operation. Age, gender, grouped Charlson comorbidity index, and IMD decile were added to the Cox regression models as determinants. Operations were followed up from the date of primary DD operation until first re-operation on the same hand, death, loss-to follow-up, or end of the study period.

Except for ethnicity, missing data were less than 1% and considered to be ‘missing completely at random’. Therefore, records with missing data were excluded from the analysis and we utilised a complete cases analysis approach. Statistical analyses were performed using Stata MP (StataCorp. 2017. Stata Statistical Software: v15. College Station, TX). Any absolute value between 0 and 5 was suppressed at the reporting stage to comply with the HES and ONS disclosure policies^14^.

### Patient and public involvement

This study addresses one of the top 10 priorities for clinical research in hand and wrist surgery, as determined by the James Lind Alliance and British Society for Surgery of the Hand Priority Setting Partnership^8^.

### Ethical approval

The study was approved by the University of Oxford Research Services (project ID 12787), and the NHS Data Access Advisory Group. Ethical approval was not required. It was conducted in accordance with the NHS Digital data sharing agreement (DARS-NIC-29827-Q8Z7Q) and registered at ClinicalTrials.gov (NCT03573765).

## Results

### Participants

**Figure 2:**
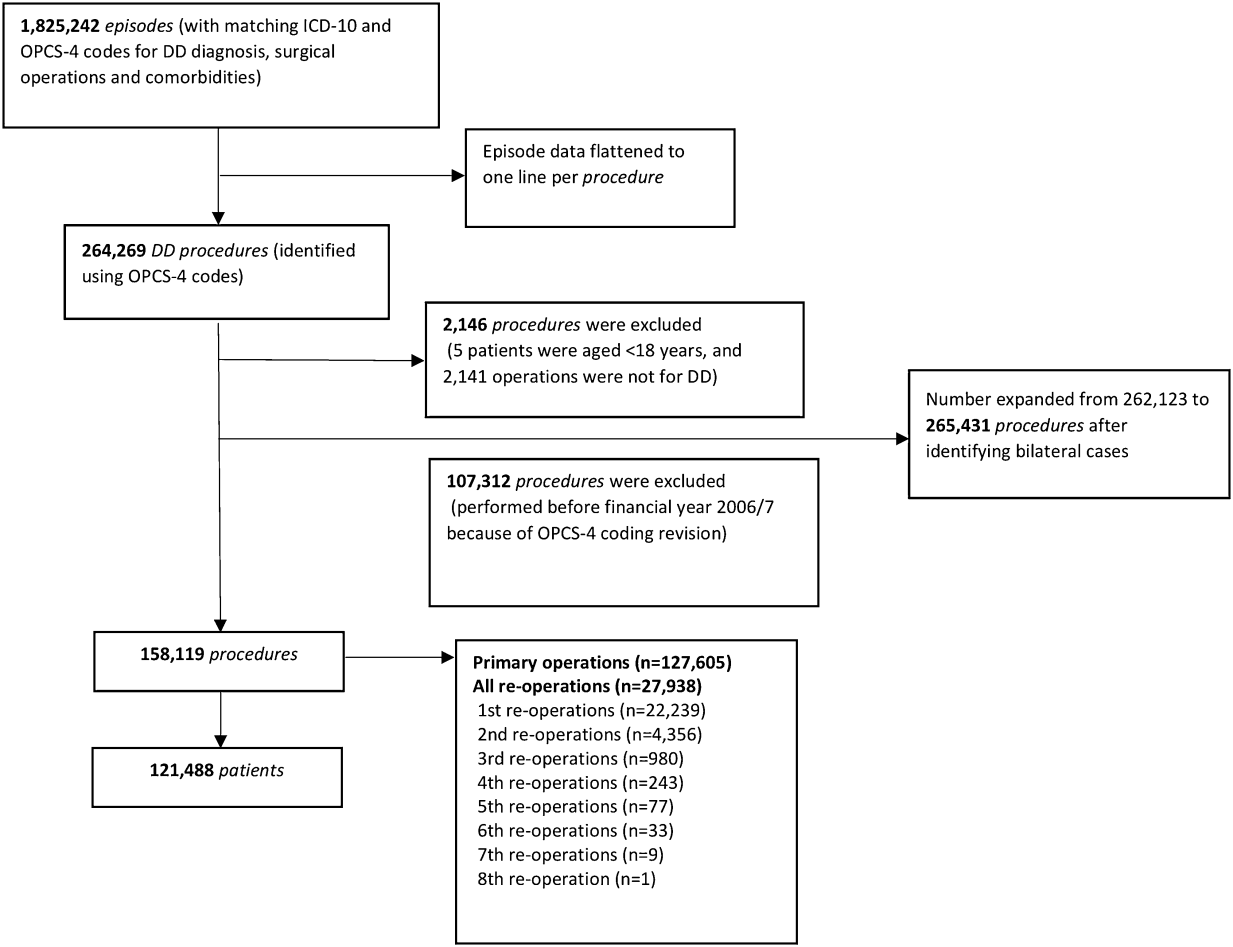
Participant flow diagram.

Between 1 April 2007 and 31 March 2017, 158,119 DD surgical procedures in 121,488 adult patients were included in the analysis. Figure 2 shows participant flow diagram at each stage of the study.

### Basic demographics

**Figure 3:**
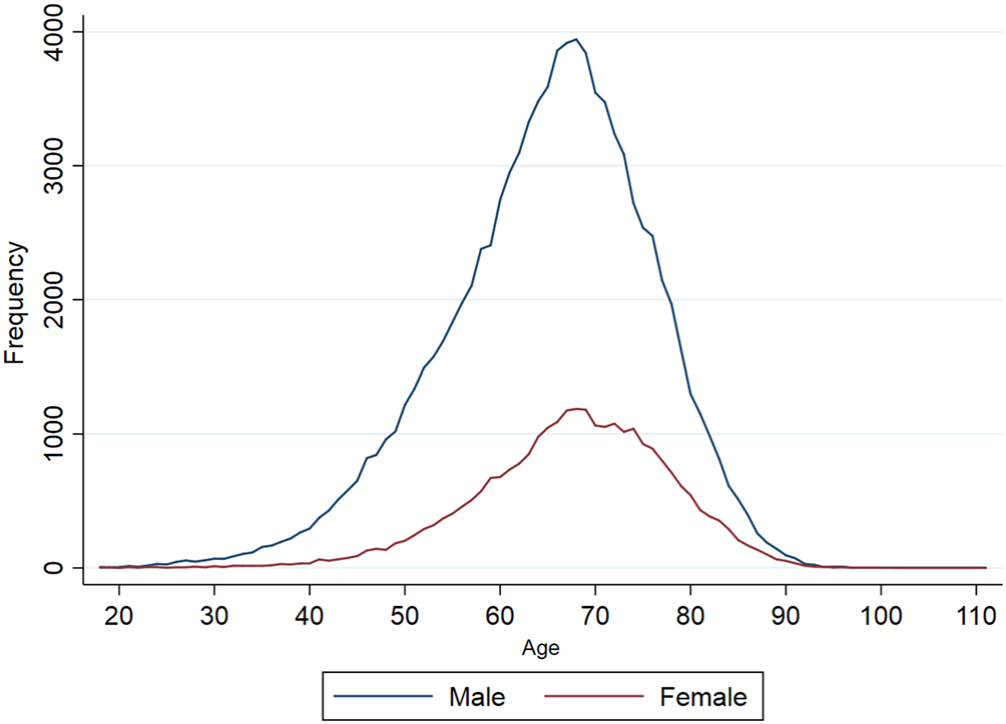
The age distribution across both genders in patients who had DD surgery in England between 1st April 2007 and 31st March 2017.

The mean (SD) age of patients at the time of surgery was 65.5 (10.67) years. The majority of patients were male (n = 94,458/121,422, 77.80%). The age distribution of male and female patients was similar (Fig. 3). 99.6% of patients were of white ethnic background (Supplementary Table 3). DD surgery was more commonly performed in the less deprived half of the population (Supplementary Table 4). The median (interquartile range (IQR)) Charlson comorbidity index of patients undergoing DD surgery was 0 (0–1).

### Main results

#### Local complications

The overall cumulative incidence of any serious local complication requiring hospital admission after DD surgery at 30 days was 1.1% (95% CI 1.0–1.1) and at 90 days was 1.2% (95% CI 1.1–1.2) (Table 1). Importantly, the overall cumulative incidence of finger amputation was 0.5% (95% CI 0.5–0.5) and 0.6% (95% CI 0.5–0.6) at 30 and 90 days post-surgery respectively. The overall cumulative incidence of finger amputation was significantly lower in primary DD surgery compared to first re-operation surgery (Table 2) at 90 days (0.3% vs. 1.5%; p < 0.0001, Chi-squared test). Remarkably, first re-operation using LF surgery after primary DF carried an extremely high cumulative incidence of finger amputation at 90 days of 8% (95% CI 4.8–13.3).

**Table 1:** Cumulative incidence of serious local complications within 30 and 90 days of all DD surgery in 150,833^a^ observations at risk.

Complication	No. of events	Cumulative incidence (% (95% CI))
**Surgical site infection (SSI)**		
30 days	51	0.03 (0.02–0.04)
90 days	59	0.04 (0.03–0.03)
**Wound dehiscence (WD)**		
30 days	76	0.05 (0.04–0.06)
90 days	79	0.05 (0.04–0.06)
**Wound complications (SSI and WD)**		
30 days	127	0.08 (0.06–0.10)
90 days	138	0.09 (0.07–0.09)
**Neurovascular injury**		
30 days	598	0.40 (0.36–0.43)
90 days	604	0.40 (0.37–0.43)
**Tendon injury**		
30 days	21	0.01 (0.01–0.02)
90 days	23	0.02 (0.01–0.02)
**Finger amputation**		
30 days	764	0.51 (0.47–0.54)
90 days	837	0.55 (0.52–0.59)
**Any local complication**		
30 days	1,637	1.09 (1.03–1.14)
90 days	1,740	1.15 (1.10–1.21)

^a^Denominator after excluding operations with missing laterality code & those with < 90 days postoperative follow up = 7286/158,119 (4.6%).

**Table 2:** Cumulative incidence of finger amputation within 90 days of DD surgery.

Primary operation	First re-operation	No. of events	No. of operations	Cumulative incidence % (95% CI)
PNF	–	22	11,271	0.20 (0.13–0.30)
LF	–	328	108,311	0.30 (0.27–0.34)
DF	–	18	4237	0.42 (0.27–0.67)
Total primary operations		368	123,819	0.30 (0.27–0.33)
PNF	PNF	< 6^a^	< 650	0.47 (0.15–1.46)
	LF	13	1534	0.85 (0.49–1.46)
	DF	< 6^a^	< 150	0.68 (0.10–4.83)
LF	PNF	14	966	1.45 (0.86–2.45)
	LF	240	15,739	1.53 (1.34–1.73)
	DF	26	2151	1.21 (0.82–1.78)
DF	PNF	0	21	0
	LF	15	187	8.02 (4.84–13.31)
	DF	< 6^a^	< 150	2.03 (0.65–6.29)
Total first re-operations		315	21,531	1.46 (1.31–1.63)

^a^Counts of 5 or less are suppressed to prevent secondary patient identification, as per NHS Digital and ONS guidance.

#### Serious systemic complications

Overall, the cumulative incidence of any serious systemic complication after DD surgery was lower than that of serious local complications, with the overall cumulative incidence of serious systemic complications being 0.3% (95% CI 0.3–0.3) at 30 days and 0.8% (95% CI 0.7–0.8) at 90 days (Table 3). Lower respiratory tract infection (LRTI) was the most commonly observed serious systemic complications, occurring in 0.2% (95% CI 0.2–0.2) at 90 days.

**Table 3:** Cumulative incidence of serious systemic complications and all-cause mortality within 30 and 90 days of all DD surgery in 153,376^a^ observations at risk.

Complication	No. of events	Cumulative incidence (% (95% CI))
**Lower respiratory tract infection**		
30 days	125	0.08 (0.07–0.1)
90 days	333	0.22 (0.20–0.24)
**Urinary tract infection**		
30 days	89	0.06 (0.05–0.07)
90 days	222	0.14 (0.13–0.17)
**Myocardial infarction**		
30 days	50	0.03 (0.02–0.04)
90 days	120	0.08 (0.07–0.09)
**Acute kidney injury**		
30 days	52	0.03 (0.03–0.04)
90 days	138	0.09 (0.08–0.11)
**Stroke (excluding mini-stroke) event**		
30 days	40	0.03 (0.02–0.04)
90 days	128	0.08 (0.07–0.10)
**Pulmonary embolism**		
30 days	23	0.02 (0.01–0.02)
90 days	60	0.04 (0.03–0.05)
**All-cause mortality**		
30 days	61	0.04 (0.03–0.05)
90 days	199	0.13 (0.11–0.15)
**Any serious systemic complication**		
30 days	440	0.29 (0.26–0.32)
90 days	1200	0.78 (0.74–0.83)

^a^Denominator after excluding procedures with < 90 days postoperative follow up = 4743/158,119 (3%).

### Self-controlled cases series (SCCS)

#### SCCS cohort characteristics

Characteristics of each nested adverse event cohort are detailed in Supplementary Table 5.

#### Association between DD surgery and serious systemic adverse events

Table 4 summarises the incidence rate ratio (95% CI) for each SCCS cohort in the 30 and 90 day postoperative periods. First primary DD surgery was associated with a doubling of risk of AKI (IRR = 2.00, 95% CI 1.14–3.53) at 30 days after surgery, and over 50% increased risk (IRR = 1.54, 95% CI 1.11–2.14) at 90 days after surgery. Similarly, there was an increased risk of MI by almost threefold (IRR = 2.90, 95% CI 1.41–5.95) at 30 days after surgery and by 2.5-fold (IRR = 2.50, 95% CI 1.66–3.77) at 90 days after surgery. The risk of LRTI was also increased by almost 30% in the 90 days after DD surgery (IRR = 1.26, 95% CI 1.03 to 1.56) but not at 30 days (IRR = 1.18, 95% CI 0.83–1.69).

**Table 4:** Incidence rate ratio (IRR) and 95% CI for serious systemic adverse events within 30 and 90 days of first primary DD surgery compared to the baseline period.

Complication	Incidence rate ratio (IRR) (95% CI) for 30-day period	Incidence rate ratio (IRR) (95% CI) for 90-day period
Lower respiratory tract infection	1.18 (0.83–1.69)	**1.26 (1.03–1.56)**
Urinary tract infection	1.10 (0.75–1.63)	1.24 (0.97–1.59)
Stroke (excluding mini-stroke)	0.86 (0.50–1.47)	1.21 (0.88–1.67)
Acute kidney injury	**2.00 (1.14–3.53)**	**1.54 (1.11–2.14)**^**a**^
Myocardial infarction	**2.90 (1.41–5.95)**	**2.50 (1.66–3.77)**
Pulmonary embolism	1.11 (0.45–2.73)	1.51 (0.89–2.56)^b^

Values in bold type represent statistically significant increased risks.

^a^Age groups < 70 were merged as group 1 and the last categories were grouped as 85 + , reference category = 70–74 age group.

^b^Age groups < 65 were merged as group 1 and the last categories were grouped as 90 + , reference category = 65–69 age group.

#### Stratified analyses

The effect of type of surgery, stratified by usual anaesthesia method, was examined by grouping PNF (performed exclusively under local anaesthesia) with LF and DF (usually performed under general or regional anaesthesia) (Supplementary Table 6). PNF was not associated with any serious systemic adverse event, whereas LF or DF were associated with MI at 30 days, and MI, AKI and LRTI at 90-days following surgery.

Gender had no significant interaction with any adverse event except for UTI, where it had a borderline significant interaction (p = 0.05). To explore the exact effect of gender in UTI, stratified analyses were conducted. Male patients had a 41% increased risk of having UTI 90 days after DD surgery (IRR = 1.41, CI 1.06–1.88), whilst females had no such excess risk of UTI post-operatively (IRR = 0.85, CI 0.52–1.40).

#### Risk of re-operations following primary DD surgery

**Figure 4:**
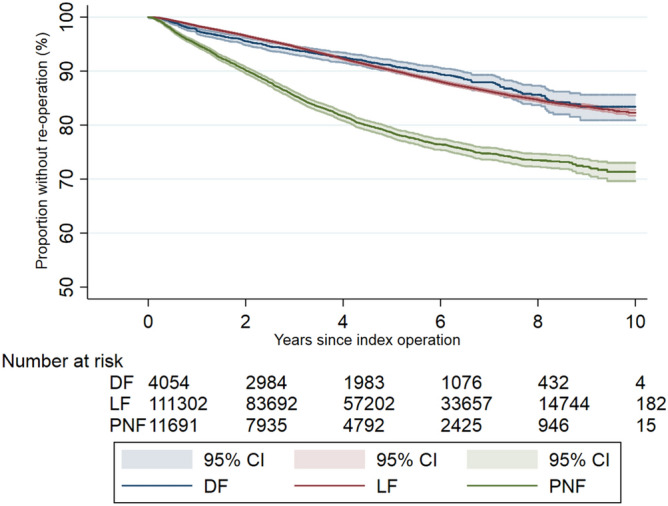
Kaplan–Meier survival estimate of time-to-first DD reoperation after primary PNF, LF and DF.

Figure 4 shows the Kaplan Meier survival curve of time to first re-operation after the three types of primary DD surgery. The overall 10-year probability of having re-operation was 19.5% (95% CI 18.9–20.1) after primary LF; 18.2% (95% CI 15.7–21.1) after primary DF, and 33.7% (95% CI 31.4–36.2) after primary PNF.

Higher age at time of primary DD operation carried a lower risk of re-operation by 3% per year of age (HR = 0.967, 95% CI 0.966–0.969) (Table 5). Men had a lower risk of re-operations compared to women (HR = 0.951, 95% CI 0.911–0.995). The risk of re-operations was around 60% lower for LF and DF compared to PNF. Lower Charlson comorbidity index was associated with a higher risk of DD re-operation. Similarly, less deprivation, as reflected by a lower IMD, was associated with higher risk of DD re-operation. For example, being in the IMD decile “least deprived 10%” was associated with increased risk of DD re-operation by 19% compared to being in the IMD decile “most deprived 10%” (HR = 1.186, 1.083–1.294).

**Table 5:** Summary of Cox regression analysis of time-to-first re-operations after primary DD surgery.

Predictors	Hazard ratio (HR), 95% CI for all primary DD operations
Age at time of operation	**0.967 (0.966–0.969)**^**a**^
**Gender**	
Women	1(base)
Men	**0.951 (0.911–0.995)**
**Type of primary surgery**	
PNF	1 (base)
LF	**0.419 (0.399–0.440)**
DF	**0.388 (0.346–0.434)**
**Grouped Charlson comorbidity index**	
GCCI (2 or more)	1 (base)
GCCI (1)	1.085 (1.012–1.164)
GCCI (0)	**1.175 (1.107–1.248)**
**Index of multiple deprivation deciles**	
Most deprived 10%	1 (base)
More deprived 10–20%	1.051 (0.954–1.159)
More deprived 20–30%	**1.103 (1.004–1.211)**
More deprived 30–40%	**1.103 (1.002–1.206)**
More deprived 40–50%	1.090 (0.995–1.192)
Less deprived 40–50%	**1.135 (1.036–1.238)**
Less deprived 30–40%	**1.129 (1.031–1.231)**
Less deprived 20–30%	**1.188 (1.088–1.295)**
Less deprived 10–20%	**1.186 (1.085–1.293)**
Least deprived 10%	**1.186 (1.083–1.294)**

Values in bold type represent statistically significant increased risks.

^a^Per increase of 1 year from age 18 (base).

## Discussion

The results of this study show that surgery for Dupuytren’s disease performed in the NHS in England was safe, with a low rate of serious local and systemic complications within 30 and 90 days. However, there is high rate of amputation within 90 days following re-operation, particularly limited fasciectomy following a dermofasciectomy. This reflects the increased risk of surgical disruption to the digital arteries when dissecting through a scarred bed, and must be discussed with patients before undertaking such procedures.

We used a self-controlled case series design to ascertain that serious systemic complications including AKI, MI, LRTI, and UTI in males were associated with surgery for DD. In addition, these relationships persisted when analysing procedures routinely performed under general or regional anaesthesia. The patients within the nested complication cohorts were generally older, had more comorbidities, and resided in areas with a higher index of multiple deprivation. These data should prompt more surgeons to consider undertaking these procedures under local anaesthesia, especially in higher risk patients^15^.

The interaction of DD surgery and its complications with social deprivation, as measured by IMD, is interesting. A recent report from NHS Health Scotland^16^ on the impact of living in a deprived area on health, found that people who live in more deprived areas are more likely to have worse health status, especially in relation to cardiovascular disease. Risk factors for cardiovascular disease, such as smoking and hypercholesterolaemia, are also risk factors for the development of DD^17,18^. It would therefore be expected that DD surgery would be more common in people living in areas of social deprivation. However, our results show that DD surgery is actually less common in this patient group, which may indicate difficulties with accessing appropriate hand surgery provision, or perhaps early mortality. Despite this, patients living in areas with more deprivation were seen to have more complications than those from areas with less social deprivation.

Our results also demonstrate that the 10-year risk of re-operation on the same hand is around 20% following either limited fasciectomy or dermofasciectomy, and around 33% following percutaneous needle fasciotomy. Dermofasciectomy is a more invasive procedure, usually reserved for patients with major skin involvement, and has previously been described to have an 11.6% recurrence rate at 5.8 years^19^. Limited fasciectomy is the most commonly performed operation for DD, and has recurrence rates in randomised controlled trials of 20–32%^20,21^, whereas recurrence after PNF is known to be higher, up to 85% at 5 years.^21^ These data, which include all NHS England patients over a 10-year period, will allow clinicians and patients to make better informed choices regarding their personalised treatment option.

The strengths of our study come from analysing a large nationwide dataset including all patients treated in the NHS in England, with a long follow-up period. This includes patient groups such as the elderly or those with multiple comorbidities who are often excluded from randomised controlled trials. Our results are therefore generalisable to the population as a whole. Serious systemic complications were evaluated using a self-controlled case series design that has not been previously used to evaluate adverse events after surgery. The SCCS design has advantages over traditional epidemiologic studies in that it intrinsically controls for time invariant confounders by comparing cases with ‘ideal’ controls, which are the cases themselves. This methodology should be applied in the future to a wide spectrum of surgical procedures.

This study has some weaknesses. DD surgery performed in private healthcare centres that are not funded by the NHS are not captured in this analysis as the HES APC dataset only covers NHS hospitals and and treatment in independent practices funded by the NHS, a minority of patients will not be captured in our dataset. Around 11% of the UK population are estimated to hold private healthcare insurance. Many minor local complications, such as minor wound infections, would not be recorded in the HES APC dataset. This means our analyses are inherently limited to serious post-operative complications warranting readmission. However, this information is valuable to patients and policymakers, as such serious complications are likely to have a more major effect on quality of life, and lead to increased healthcare costs. Similarly, our definition of re-operation on the same hand is not identical to the recently agreed consensus for a definition of recurrence^22^. It is possible that some re-operations were for extension of disease to another finger, and that patients with true recurrence decided to not have repeat surgery.

Our data provide useful information regarding complications and re-operation rates after DD surgery. There are conflicting advantages and disadvantages of each surgical treatment option that clinicians must communicate to their patients. On the one hand, PNF is minimally invasive and carries a lower risk of serious complications. However, this comes at the cost of an increased rate of re-operation. This is of interest to patients, clinicians and policymakers treating this very common condition. Further research should examine whether more widespread use of local anaesthesia in the surgical management of DD can reduce systemic complications. Furthermore, linking primary and secondary care datasets would produce a more complete picture of local complication rates. This will allow a detailed analysis of the costs associated with DD surgery. Combined with routinely collected patient reported outcome measures, utilising the UK Hand Registry, this will provide evidence of cost effectiveness for the alternative DD surgical treatments, and guide decision making by clinicians, policymakers, and patients.

## Supplementary information

Supplementary file 1

## Data Availability

The study is based on NHS Hospital Episode Statistics data and were provided within the terms of an NHS Digital data sharing agreement. The data do not belong to the authors and may not be shared by the authors, except in aggregate form for publication. Data can be obtained by submitting a research request through the NHS Digital Data Access Request Service.
