# Supplementary material for: Serious complications and risk of re-operation after Dupuytren’s disease surgery: a population-based cohort study of 121,488 patients in England
Source: Sci Rep. 2020 Oct 5;10:16520. doi: 10.1038/s41598-020-73595-y (PMC7536429; doi:10.1038/s41598-020-73595-y)
Supplement: Supplementary file 1 [file 41598_2020_73595_MOESM1_ESM.pdf]

Supplementary file

Serious complications and risk of re-operation after Dupuytren's disease surgery: a population-based cohort study of 121,488 patients in England

Authors

Osaid Alser,[1] MSc by Research Student in Musculoskeletal Sciences
Richard S Craig,[1] Royal College of Surgeons and National Joint Registry Research Fellow
Jennifer C E Lane,[1] Versus Arthritis Clinical Research Fellow, MRC Doctoral Training Fellow
Albert Prats-Uribe,[2] DPhil Student in Musculoskeletal Sciences
Danielle E Robinson,[2] Research Associate in Pharmaco-epidemiology
Jonathan L Rees,[1] Professor of Orthopaedic Surgery and Musculoskeletal Science
Dani Prieto-Alhambra[2*], Professor of Pharmaco- and Device Epidemiology
Dominic Furniss,[1,3*] Associate Professor of Plastic Surgery

Authors affiliation

1. Oxford NIHR Musculoskeletal Biomedical Research Unit, Nuffield Department of Orthopaedics, Rheumatology and Musculoskeletal Sciences, Nuffield Orthopaedic Centre, University of Oxford, Windmill Road, Oxford, OX3 7LD, UK
2. Centre for Statistics in Medicine, NDORMS, University of Oxford, Windmill Road, Oxford, OX3 7LD, UK
3. Department of Plastic and Reconstructive Surgery, Nuffield Orthopaedic Centre, Windmill Road, Oxford, OX3 7HE, UK
   * These authors co-directed this work

Correspondence to Dominic Furniss, NDORMS, Oxford University, Botnar Research Centre, Oxford, OX3 7LD, UK. ORCID ID (https://orcid.org/0000-0003-2780-7173) Twitter handle (@dominicfurniss) dominic.furniss@ndorms.ox.ac.uk
++441865227233

Supplementary Table S1: DD diagnostic (ICD v10) and operative codes (OPCS v4.4, 4.5, 4.6, 4.7) for surgical treatment

Grouping	Condition	OPCS-4 code		ICD-10 code
	Palmar fascial fibromatosis (DD)	T521		M720
LF	Palmar fasciectomy			
	Revision of palmar fasciectomy	T522		
	Digital fasciectomy	T525		
	Revision of digital fasciectomy	T526		
DF	Dermofasciectomy	T561		
	Revision of dermofasciectomy	T562		
PNF	Division of palmar fascia	T541	Linked with (+)	M720
	Other specified division of fascia	T548		
	Unspecified division of fascia	T549		
	Other specified release of fascia	T558		
	Unspecified release of fascia	T559		
LF	Other specified excision of other fascia	T528		
	Unspecified excision of other fascia	T529		
	Other specified other excision of other fascia	T568		
	Unspecified other excision of other fascia	T569		
Anatomical codes	Hand NEC	Z894		
	Thumb NEC	Z895		
	Finger NEC	Z896		
	Multiple digits of hand NEC	Z897		
	Skin of finger	Z503		
	Bilateral operation	Z941		
	Right sided operation	Z942		
	Left sided operation	Z943		
	Unilateral sided operation	Z944		
	Specified laterality NEC	Z948		
	Laterality NEC	Z949		

Supplementary Table S2a: DD diagnostic (ICD v10) and operative codes (OPCS v4.4, 4.5, 4.6, 4.7) for local postoperative complications

Grouping	Condition	OPCS-4 code	Linking	ICD-10 code
Local complications	Wound complications: surgical site infection (SSI) that required wound debridement	(Z894 or Z895 or Z896 or Z897 or Z898 or Z503) + (T963 or T964 or T968 or T969)	Linked with (+)	T814 or L025
	Wound complications: wound dehiscence that required closure	(Z894 or Z895 or Z896 or Z897 or Z898 or Z503) + (S604 or S422 or S423 or S424)		
	Neurovascular injury (that required repair)	(Z894 or Z895 or Z896 or Z897 or Z898 or Z503) + (A641 or A642 or A643 or A644 or A648 or A649 or A621 or A622 or A623 or A624 or A625 or A626 or A627 or A628 or A629 and A631 or A632 or A638 or A639 or A64 or Z097)	Linked with (+)	T810 or T812
	Tendon injury (that required tendon repair)	(Z894 or Z895 or Z896 or Z897 or Z898 or Z503) + (T671 or T672 or T673 or T674 or T675 or T676 or T678 or T679)		
	Finger amputation	X082 or X083 or X084		

Supplementary Table S2b: DD diagnostic (ICD v10) codes for serious systemic postoperative complications

Grouping	Condition	ICD-10 code
Serious systemic complications	Stroke (excluding mini-stroke)	I60X or I61X or I62X or I63X or I64X
	Lower respiratory tract infection (LRTI): (pneumonia, aspiration pneumonia, lung abscess with pneumonia, COPD exacerbation)	J12X or J13X or J14X or J15X or J16X or J18x J20X or J22 or J86X or J440 or J851 or J690
	Acute myocardial infarction (AMI)	I21X or I22X
	Pulmonary embolism (PE)	I26X
	Urinary tract infection (UTI)	N300 or N308 or N309 or N390 or N10
	Acute renal failure (ARF)	N170 or N171 or N172 or N178 or N179 or N990
	Death	Extracted from the Office for National Statistics (ONS) mortality data

Supplementary Table S3: Ethnicity grouping and frequency of each ethnic category

Grouped ethnicity	Original categories	Frequency*
**White**	British (White), Irish (White), any other white background	105,822 (98.58%)
**Asian**	Indian, Indian (Asian or Asian British), Bangladeshi, Bangladeshi (Asian or Asian British), Pakistani, Chinese, Chinese (other ethnic group), any other Asian background	589 (0.55%)
**Black**	Black (Caribbean), Black (African), Caribbean (Black or Black British), African (Black or Black British), any other Black background	357 (0.33%)
**Mixed ethnicity**	Any other mixed background , White and Black Caribbean (Mixed), White and Black African (Mixed), White and Asian (Mixed)	159 (0.15%)
**Any other ethnicity**	Any other ethnicity	423 (0.39%)
**Unknown ethnicity (missing)**	Not given, not stated, not known	14,138 (11.64%)
**Total**		121,488 (100%)

*Denominator for all groups except missing=107,350 (total without missing observations)

Supplementary Table S4: Index of multiple deprivation (IMD) deciles

IMD deciles	Number of patients	Frequency (%)*
**Least deprived 10% (Decile 1)**	13,715	11.4
**Less deprived 10-20% (Decile 2)**	14,383	11.9
**Less deprived 20-30% (Decile 3)**	14,764	12.3
**Less deprived 30-40% (Decile 4)**	14,442	12.0
**Less deprived 40-50% (Decile 5)**	13,564	11.3
**More deprived 40-50% (Decile 6)**	12,349	10.3
**More deprived 30-40% (Decile 7)**	10,958	9.1
**More deprived 20-30% (Decile 8)**	9,686	8.0
**More deprived 10-20% (Decile 9)**	8,520	7.1
**Most deprived 10% (Decile 10)**	7,983	6.6
**Missing**	1,124	0.93**
**Total**	121,488	100

*Denominator=120,364 (total without missing IMD deciles)
**Denominator=121,488

Supplementary Table S5: Characteristics of patients in each SCCS cohort

	LRTI cohort (n=429)	UTI cohort (n=300)	Stroke cohort (n=180)	AKI cohort (n=178)	MI cohort (n=140)	PE cohort (n=67)
**Index surgery**						
PNF, n (%)	58 (13.52)	31 (10.33)	24 (13.33)	26 (14.61)	19 (13.57)	<6**
LF, n (%)	357 (83.22)	259 (86.33)	151 (83.89)	149 (83.71)	116 (82.86)	58 (86.57)
DF, n (%)	14 (3.26)	10 (3.33)	<6**	<6**	<6**	<6**
**Age at time of surgery, mean (SD)**	70.31 (9.89)	70.94 (10.56)	69.89 (9.01)	70.28 (10.50)	69.98 (9.79)	68.45 (9.89)
**Gender (Males), n (%)**	355 (82.75)	215 (71.67)	151 (83.89)	145 (81.46)	122 (87.14)	50 (74.63)
**Ethnicity**						
White, n (%)	392 (98.49)	260 (97.01)	159 (100)	166 (98.81)	129 (98.47)	57 (98.28)
Others, n (%)	6 (1.51)	8 (2.99)	0	<6**	<6**	<6**
Missing, n (%)	31 (7.23)	32 (10.67)	21 (11.67)	10 (5.62)	9 (6.43)	9 (13.43)
**Charlson Comorbidity Index, median (IQR)**	1 (1-3)	1 (1-3)	1 (1-2)	1 (2-5)	1 (1-3)	1 (1-2)
**IMD deciles, n (%)**						
Less and least deprived 50%	215 (50.23)	137 (45.67)	80 (52.02)	91 (51.40)	58 (41.72)	27 (40.32)
More and most deprived 50%	213 (49.76)	163 (54.34)	97 (54.79)	86 (48.58)	81 (58.27)	40 (59.66)
Missing	1 (0.23)	0	3 (1.67)	1 (0.56)	1 (0.71)	0

*Percentages for all categories were based on the total without missing as a denominator except missing category where the total was its denominator (complete case analysis)

**Counts of 5 or less are suppressed to prevent secondary patient identification, as per NHS Digital and ONS guidance.

Supplementary Table S6: Incidence rate ratio (IRR) and 95% CI for serious systemic adverse events within 30 and 90 days of each first primary DD surgery (PNF vs. LF or DF) compared to each adverse event in the baseline period

Complication	Type of surgery	Incidence rate ratio (IRR) (95% CI) for 30-day period	Incidence rate ratio (IRR) (95% CI) for 90-day period
**Lower respiratory tract infection**	PNF	0.46 (0.18-1.21)	0.89 (0.49-1.61)
	LF or DF	1.40 (0.93-2.10)	**1.30 (1.03-1.63)**
**Urinary tract infection**	PNF	0.67 (0.11-3.99)	0.82 (0.38-1.74)
	LF or DF	1.10 (0.72-1.65)	1.28 (0.98-1.67)
**Stroke (excluding mini-stroke)**	PNF	4.00 (0.45-35.79)	1.30 (0.52-3.27)
	LF or DF	0.60 (0.32-1.11)	1.10 (0.78-1.57)
**Acute kidney injury**	PNF	1.33 (0.30-5.96)	2.02 (0.86-4.72)
	LF or DF	1.81 (0.95-3.45)	**1.49 (1.03-2.13)**
**Myocardial infarction**	PNF	4.00 (0.45-35.79)	1.83 (0.68-4.96)
	LF or DF	**2.36 (1.17-4.78)**	**2.24 (1.49-3.35)**
**Pulmonary embolism**	PNF	*	1 (0.14-7.10)
	LF or DF	1.57 (0.61-4.05)	1.59 (0.93-2.72)

*The incidence of adverse event was too low to allow the SCCS model to converge
